# Anton's Syndrome due to Bilateral Ischemic Occipital Lobe Strokes

**DOI:** 10.1155/2014/474952

**Published:** 2014-11-03

**Authors:** Sanela Zukić, Osman Sinanović, Lejla Zonić, Renata Hodžić, Svjetlana Mujagić, Edina Smajlović

**Affiliations:** ^1^Department of Neurology, University Clinical Center Tuzla, Trnovac bb, 75000 Tuzla, Bosnia and Herzegovina; ^2^Department of Radiology, University Clinical Center Tuzla, 75000 Tuzla, Bosnia and Herzegovina; ^3^Justice Execution Institution, 83233 Bernau, Germany

## Abstract

We present a case of a patient with Anton's syndrome (i.e., visual anosognosia with confabulations), who developed bilateral occipital lobe infarct. Bilateral occipital brain damage results in blindness, and patients start to confabulate to fill in the missing sensory input. In addition, the patient occasionally becomes agitated and talks to himself, which indicates that, besides Anton's syndrome, he might have had Charles Bonnet syndrome, characterized by both visual loss and hallucinations. Anton syndrome, is not so frequent condition and is most commonly caused by ischemic stroke. In this particular case, the patient had successive bilateral occipital ischemia as a result of massive stenoses of head and neck arteries.

## 1. Introduction

Visual anosognosia, or denial of loss of vision, which is associated with confabulation in the setting of obvious visual loss and cortical blindness is known as Anton's syndrome [1]. Originally, the syndrome is named by Gabriel Anton, who described patients with objective blindness and deafness showing a lack of self-perception of their deficit. Later Joseph Babinski used the term anosognosia to describe this phenomenon [2, 3]. Bilateral occipital brain damage results in blindness; however, patients start to confabulate to fill in the missing sensory input.

Why patients with Anton's syndrome deny their blindness is unknown, although there are many theories. Although visual anosognosia is frequently believed to represent cortical phenomenon, it is probably more often caused by parietal white matter injury leading to a disconnection syndrome [4, 5].

In this paper, we present a case of patient with Anton's syndrome due to bilateral occipital ischemic lesions as a result of massive stenoses of head and neck arteries.

## 2. Case Presentation

**Figure 1:**
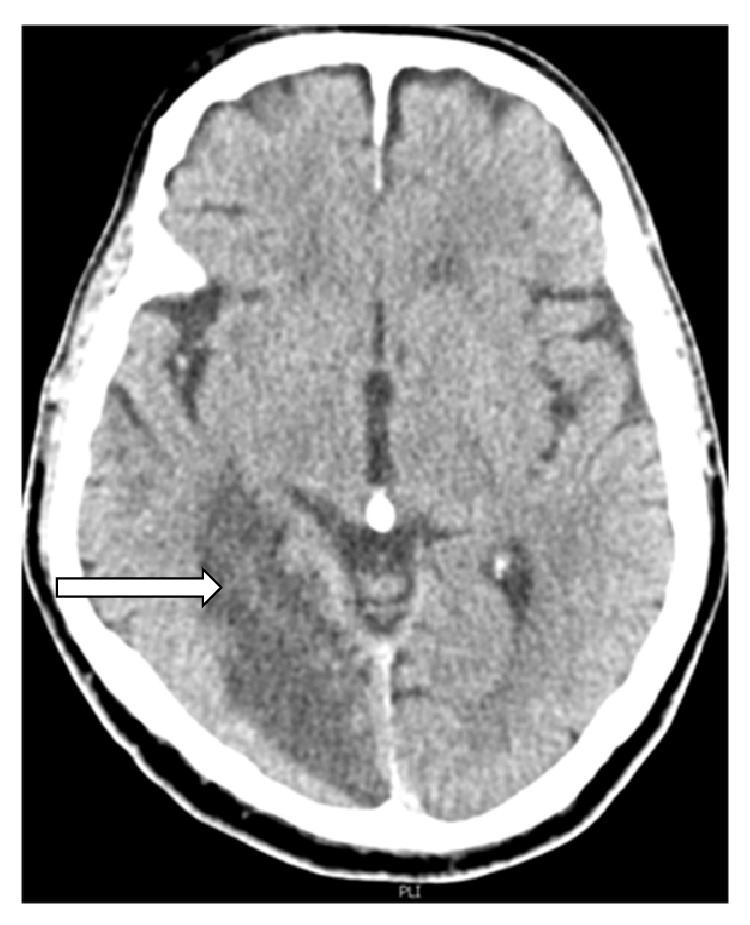
An axial CT image of the brain shows classic nonhemorrhagic cerebral infarction in the right posterior cerebral artery territory (*white arrow*).

**Figure 2:**
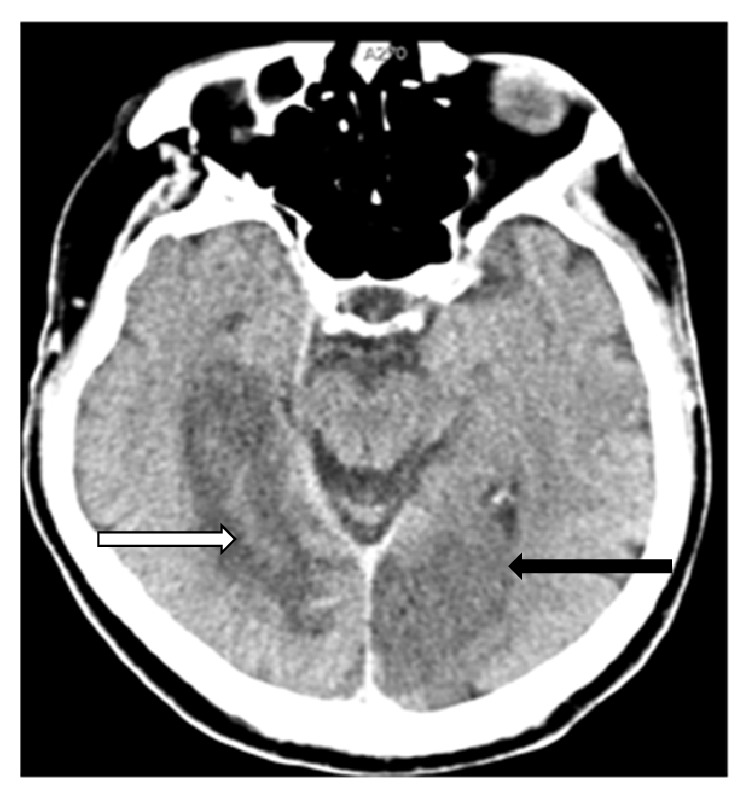
An axial CT image of the brain shows subacute cerebral infarction in the right posterior cerebral artery territory (*white arrow*) and new acute cerebral infarction in the left posterior cerebral artery territory (*black arrow*).

A 76-year-old man has been admitted to the Neurology Department of University Clinical Center Tuzla due to a sudden and moderate paresis of the left hand and left leg and impaired speech with dysarthria and without elements of anosognosia or unilateral neglect. Previous medical history revealed longstanding hypertension, diabetes, and atrial fibrillation. The Glasgow coma scale (GCS) score was 15 out of 15. Neurological examination revealed left homonymous hemianopsia, central type facial palsy, and paresis of left extremities. He was eupneic, afebrile, and hypertensive. He also had a systolic murmur over the right carotid artery. Color Doppler of the neck vessels performed immediately after the admission showed a complete occlusion of the left internal carotid artery (ICA) and left vertebral artery (VA), also moderate stenosis of the right ICA and significant stenosis of the right VA, with atherosclerotic plaques on all other blood vessels. Urgent computed tomography (CT) of the brain revealed an ischemic lesion in the right temporooccipital region (Figure 1). Soon after the admission, the patient develops a new neurological deficit of right sided paresis. Follow-up CT scans revealed a newly developed left occipital acute ischemia (Figure 2).

**Figure 3:**
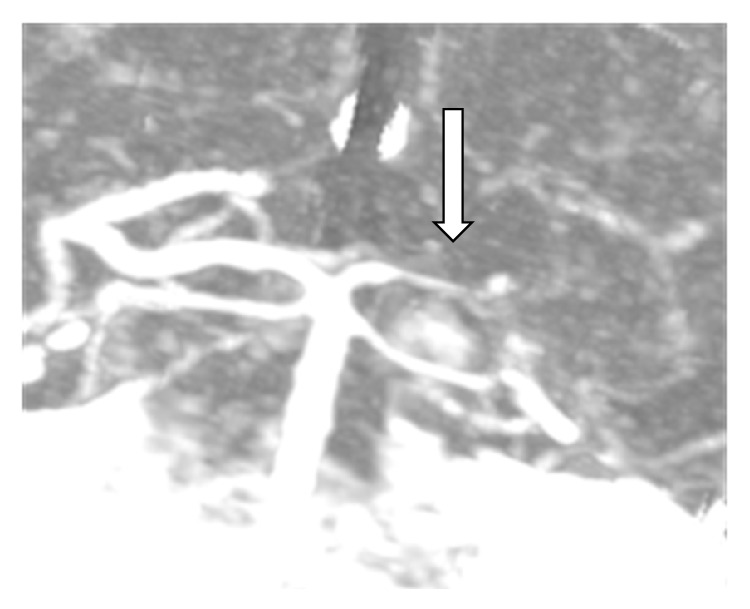
CT angiography shows gracile flow of the right posterior cerebral artery with narrowing of the P1 segment (*white arrow*).

CT angiography confirmed the ultrasound findings along with an incipient stenosis of the left subclavian artery. The right posterior cerebral artery showed a gracile flow with narrowing in the middle of the artery (Figure 3), with atherosclerotic changes in the remaining blood vessels of the head and neck.

**Figure 4:**
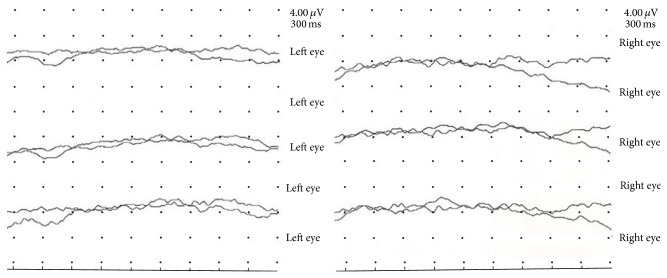
Visual evoked potential, absence of visual evoked response on both eyes.

Newly developed neurological deficit also included a gradual loss of vision, due to bilateral occipital lesion. Ocular movements and pupillary reflexes were intact suggesting that anterior visual pathways were not damaged. Fundoscopy was unremarkable. The patient was not aware of the sight loss. In particular, the sight loss was observed for the first time when the patient asked for a door to be opened, even though the door was already standing wide open. When asked about the position of the door, the patient pointed to the obviously wrong direction. Also when asked to describe the attending physician, the patient provided a completely wrong visual description of the physician. In addition, he was unable to reach physician's extended hand. Despite this obvious blindness, the patient suffered a visual anosognosia, since he was unaware of his blindness and was confabulating about his surroundings when asked about it. Complete blindness was confirmed by ophthalmologist due to an absence of response to simulation of visual evoked potentials (Figure 4). The patient adamantly claimed he was able to see, despite the confirmed blindness test. Furthermore, medical staff reported that he would occasionally become agitated and talk to himself. Consequently, the patient has been treated with clopidogrel, antihypertensive, antidiabetic, and statin drugs. The drugs treatment, together with physical and speech therapy, results in an improvement of reduction in neurologic deficit. However, at the time of discharge, persistent elements of Anton's syndrome were present. The patient has been followed up as an outpatient, having a neurological improvement and being able to walk with minor help. Blindness remained permanent. One year later the patient deceased due to cardiovascular complications.

## 3. Discussion

Bilateral occipital stroke is a common cause of visual anosognosia also known as Anton's syndrome [6]; however, consecutive occipital strokes as a cause of Anton's syndrome are rather uncommon. In our patient, at admission CT of the brain revealed only ischemic lesion in the right temporooccipital region, but soon after the admission an ischemic lesion in the left occipital region has developed. Cortical blindness due to bilateral damage of the occipital lobes was most likely secondary to hypoxia, vasospasm, and cardiac embolism [7].

Confabulation is one of the important criteria of Anton's syndrome. Anton suggested that damaged visual areas are effectively disconnected from functioning areas, such as speech and language areas. In the absence of input, functioning speech areas often confabulate a response [1, 8].

Our patient showed all aspects of Anton's syndrome, visual anosognosia, and confabulation. However, medical staff also reported that he would occasionally become agitated and talk to himself, which may indicate that he might have had Charles Bonnet syndrome, characterized by both visual loss and hallucinations [9].

Bilateral cortical blindness and Anton syndrome are most commonly caused by bilateral occipital lobe lesions [1, 5, 10]. This syndrome was also reported in a few other medical conditions such as gynecological complications (preeclampsia and obstetric hemorrhage) [11], MELAS [12], trauma [13], adrenoleukodystrophy [14], hypertensive encephalopathy [15], and angiographic procedures [16].

Considering that recovery of visual function depends on the underlying etiology, in this case we could not expect the full recovery mainly because of multiple stenoses of head and neck arteries. Patient was not considered for surgical treatment because of age and other risk factors. Therefore, management was focused on secondary prevention and rehabilitation.

## 4. Conclusion

We should suspect Anton's syndrome (visual anosognosia), when the patient has denial of blindness with evidence of occipital lobe damage, or even the Charles Bonnet syndrome, which is comprised of both the visual loss and hallucinations.
